# Sublay or onlay incisional hernia repair along with abdominoplasty: which is better? Long-term results

**DOI:** 10.1007/s10029-019-01914-y

**Published:** 2019-02-25

**Authors:** A. Iljin, B. Antoszewski, T. Zieliński, A. Skulimowski, D. Szymański, J. Strzelczyk

**Affiliations:** 1 Department of Plastic, Reconstructive and Aesthetic Surgery, Medical University of Lodz, Kopcinskiego 22, 90-153 Lodz, Poland; 2 Department of General and Transplant Surgery, Medical University of Lodz, Kopcinskiego 22, 90-153 Lodz, Poland

**Keywords:** Bariatric surgery, Abdominoplasty, Sublay IHR, Onlay IHR

## Abstract

**Purpose:**

Estimation and comparison of results after incisional hernia repair (IHR) modo onlay or sublay with abdominoplasty in patients who lost the weight following Roux-en-Y Gastric Bypass (RYGB). Analysis and comparison of changes in quality of life (QL) of these patients prior to RYGB, before and after simultaneous IHR and abdominoplasty.

**Methods:**

Clinical analysis involved 40 patients with abdominal disfigurement (following RYGB and massive weight loss) after one-time IHR sublay method with abdominoplasty—group 1 or IHR onlay method with abdominoplasty—group 2. We evaluated postoperative results and long-term QL changes (DAS24, SF-36 scales).

**Results:**

We noted abnormal wound healing (2), pneumonia (3) and dysesthesia (3) in patients from group 1, and abnormal wound healing (2), seroma (2), pneumonia (2), and dysesthesia (4) in group 2. Quality of life was improved in the functional, esthetic and psychological aspects.

**Conclusions:**

One stage incisional hernia repair by onlay as well as sublay method with abdominoplasty are safe surgical methods improving the functioning of patients after major weight loss following RYGB. Sublay hernia repair and abdominoplasty was connected with longer time of the: operation, drainage, analgesic agents use, time to mobilization and to full oral diet than the onlay method. Significant improvement of the quality of life was noted after every subsequent step of surgical treatment in both groups. Reduction of the risk of BMI re-growth after bariatric surgery is related to the need for constant, specialized care for these patients at every stage of follow-up after bariatric surgery.

## Introduction

Obesity is recognized a social disease with epidemic status as over 30% of adult world population presents body mass index (BMI) exceeding 30 kg/m^2^. Bariatric surgery is a commonly performed highly effective treatment in achieving long-term weight loss and resolution of obesity-related comorbidities. However, in the majority of these patients body contour irregularities and postoperative hernias (in 10–50%) form after open bariatric procedures [1–5]. Skin redundancy at different body parts observed in more than two-thirds of patients after major weight loss, in some cases with malpositioned adipose tissue, are responsible for hygiene problems, persistent inflammatory changes inside skin folds and both for physical and psychosocial discomfort for the patients. Insufficient self-control in patients following bariatric surgery is a very common reason for their weight regain. All together these mentioned distant consequences negatively affect the quality of life (QL) in postbariatric groups [4, 6, 7]. As other authors state, the amount of weight loss is not related to the degree of improvement in persons who were previously morbidly obese, as they become less satisfied with their body image with increasing weight loss. However, favorable changes in appearance after esthetic operations following massive weight loss undoubtedly positively alter their QL. Hence, body contouring procedures as a part of multidisciplinary treatment in these patients constitute a consecutive step in their rehabilitation [8, 9]. Body dysmorphism resulting from weight loss in excess of 30% is not usually amenable to full correction by standard abdominoplasty, but resection of loose skin in the lower abdomen tends to be the first request in postbariatric subjects [10]. Abdominoplasty in bariatric patients can be performed as a single procedure, also together with body contouring surgery or with abdominal hernia repair after open bariatric procedures [3, 11–16]. Given the excellent intraoperative exposure of the abdominal wall during abdominal contouring surgery, concurrent ventral, umbilical, or inguinal hernia repair is also often performed. Despite generally higher risk of complications (seroma formation, wound infection) following simultaneous abdominal wall surgery, most of the authors reported favorable outcomes of abdominoplasty combined with hernia repair [1, 3, 13, 16]. Complex body contouring surgery in postbariatric groups after massive weight loss has been discussed regarding postoperative results and patients’ life quality, however, there are no reports on long-term observations comparing abdominoplasty along with incisional hernia repair (IHR) performed using two separate techniques. Onlay IHR is technically easier, but related to increased rate of wound complications, mesh infection, and hernia recurrence. Sublay IHR requires greater surgical expertise, and for the patients is connected with longer postoperative recovery time, but undoubtedly the advantage of this method is location of the mesh under rectus muscles providing proper blood supply [17, 18].

Therefore, the aim of our study was to evaluate early postoperative course as well as long-term results after simultaneous IHR (sublay versus onlay method) along with abdominoplasty in patients following RYGB (Roux-en-Y Gastric Bypass), and to compare their QL prior to RYGB, and before as well as after abdominal wall surgery.

## Methods

We performed a single institution cohort study. The study comprised all patients between 2009 and 2015, who underwent open RYGB for morbid obesity and then after stabilization of weight loss IHR with abdominoplasty using sublay method (group 1) or onlay method (group 2) (Table 1). The patients were divided into two groups before surgery. Incisional hernias in individuals from both groups varied in respect of size, and the selection of method for surgical repair—onlay or sublay was based on their accurate assessment done intraoperatively (Table 2). IHR and abdominoplasty were performed as a single procedure in all eligible patients, who gave their consent to such combined operation. During the first stage a cutaneosubcutaneous flap in the shape of an inverted T was typically incised along the suprafacial plane. After umbilicus translocation, the flap was dissected from the muscular aponeurosis. Then, two lateral folds were mobilized up to the costal arches. In the sublay method a posterior sheath of the abdominal rectus muscle was detached and polypropylene mesh was connected to its margins with interrupted, prolene sutures. Suction drain was inserted and put on the mesh. Muscle layer was sutured and subcutaneous drainage was applied. In the onlay method the polypropylene mesh was attached to the anterior rectus sheath with interrupted, prolene sutures, and suction drain was placed over the mesh. Reinforced polypropylene mesh was 8 cm wide in onlay IHR and 6 cm in sublay IHR. The length of the mesh depended on the size of the surgical wound/hernia (Table 2) being nearly 4 cm longer than its length, which means there was 2 cm mesh overlap both in the upper part of the wound and 2 cm in the lower part of the wound. Routine low-molecular-weight heparin as well as antibiotic perioperative prophylaxis were used in both groups.

**Table 1:** Demographic data of the analyzed groups

Number of patients	Sublay IHR	Onlay IHR	*p*
40	20	20	
Females	12	16	
Age: mean (years)	38	43.2	0.074
Age: range (years)	23–53	27–55	
Pre RYGB BMI	53.68 [46.3] (± 10.15)	46.84 [46.7] (± 4.63)	0.005
Pre IHR and abdominoplasty BMI	30 [29.7] (± 3.27)	29.56 [29.25] (± 4.64)	0.742
Post IHR and abdominoplasty BMI	28.37 [28.1] (± 3.13)	28.17 [28] (± 4.5)	0.926
Weight regain 3–5 years after RYGB	32.67 [33.3] (± 5.12)	30.16 [30.3] (± 7.64)	0.192
Interval between RYGB and IHR with abdominoplasty (months)	22 [24] (± 8)	21 [24] (± 8)	0.64
Comorbidities at the time of IHR and abdominoplasty			
Hypertension	9	7	
Disorders of the skeletal system	14	10	
Diabetes	5	3	
Respiratory system dysfunction	5	3	
Disorders of lipid metabolism	4	4	
Depression	2	2	
Fertility disorders	0	2	
Varicose veins of the lower limbs	3	2	
Hypothyroidism	3	2	
Active smokers	6	5	

The values are given following order: mean, median, standard deviation

**Table 2:** The list of comparable parameters related to IHR and abdominoplasty in the examined groups

Examined factors related to IHR and abdominoplasty	Sublay	Onlay	*p*
Duration of IHR and abdominoplasty (h)	2.77 [3] (± 0.5)	1.8 [1.75] (± 0.5)	< 0.0001
Weight of resected tissue-average (kg)	4.6 [4.75] (± 1)	3.88 [4] (± 1.45)	0.2
Incisional hernia size:			
Range (cm)	10–15	12–18	
Mean (cm)	13.2	15.6	< 0.0001
Duration of hospitalization (days)	10 [8.5] (± 3)	10.5 [5] (± 3)	0.001
Time to mobilization (days)	2.55 [3] (± 1)	2 [2] (± 0)	0.07
Duration of suction drainage (days)	7 [7] (± 2)	5 [4] (± 1)	0.003
Time to full oral diet (days)	2.75 [3] (± 1)	2.2 [2] (± 0)	0.005
Duration of analgesic agents (days)	12 [12] (± 7)	5 [4] (± 1)	0.001

All patients were followed up in the out-patient clinic on a monthly basis (during the first 6 months), than every 3 months, and later once a year during consecutive years. The follow-up period varied from 8 to 3 years depending on the time of RYGB. We analyzed and compared the postoperative course and results in patients after onlay IHR versus sublay IHR and abdominoplasty. We compared also QL of the patients from these both groups. QL survey was used to assess the physical and mental dimensions basing on the Short Form SF-36 Health Survey, and esthetic aspect was estimated using the short form of the Derriford Appearance Scale-DAS24 adopted for our postbariatric individuals. SF-36 comprises eight health concepts: physical functioning, bodily pain, role limitations due to physical health problems, role limitations due to personal or emotional problems, emotional well-being, social functioning, energy/fatigue, and general health perceptions. DAS24 is a 24-item scale measuring distress and dysfunction related to problems with general appearance, in particular the intensity of emotional response, frequency of particular behaviors and physical impact on the problem of appearance (concerning pain and functional limitation). These scales were applied in the examined groups of patients before RYGB, and then before and 3 years after IHR and abdominoplasty. Variables were tested with either Student’s *t* test (when comparing two groups with normal distribution), Mann–Whitney test (when comparing two groups with non-normal distribution). Statistical analysis was performed using IBM SPSS Statistics for Windows, Version 24.0 (IBM Corp. Armonk, NY).

## Results

Demographic data of the studied patients have been listed in Table 1, parameters related to operative procedures—sublay and onlay IHR along with abdominoplasty—and to their postoperative course in Table 2, direct and long-term surgical results in Table 3, and QL results (SF-36, DAS24) in Table 4.

**Table 3:** Results and complications after IHR and abdominoplasty in the examined patients

	Sublay	Onlay
Infection, abnormal wound healing	2	2
Seroma	0	2
Hematoma	0	0
Bronchogenic pneumonia	3	2
Vein thrombosis	0	0
Fat embolism, thromboembolism	0	0
The presence of intertrigo (under the abdomen pendulum confirmed in all patients before IHR and abdominoplasty)	0	0
Appearance of postoperative scars		
Linear–esthetic	17	18
Wide	3	2
“Dog ears”	0	0
Abdominal integument dysesthesia		
Transient	3	2
Persistent	0	2
Flaccidity in the epi- and hypogastric regions	0	0
Dysesthesia in the area innervated by the lateral cutaneous nerve of the thigh	0	0
Disfigurement of pubic hair	0	0
Umbilicus translocation behind the medial line	0	0
Umbilicus necrosis	0	0
Abdominal wall asymmetry	0	0
Hernia recurrence	0	0

**Table 4:** Evaluation of QL (SF-36, DAS24) in patients from the examined group

	Sublay	Onlay	*p*
Pre RYGB SF-36	138 [146] (± 29)	136 [140] (± 29)	0.6
Pre RYGB SF-36 (physical)	81 [85] (± 15)	79 [82] (± 12)	0.43
Pre RYGB SF-36 (mental)	58 [66.5] (± 24)	57 [62] (± 11)	0.48
Pre IHR and abdominoplasty SF-36	48 [49] (± 12)	47 [49] (± 11)	0.72
Pre IHR and abdominoplasty SF-36 (physical)	29 [34] (± 12)	28 [30] (± 9)	0.31
Pre IHR and abdominoplasty (mental)	19 [19.5] (± 6)	19 [19] (± 5)	0.24
Post IHR and abdominoplasty SF-36	14 [12.5] (± 8)	16 [17] (± 8)	0.18
Post IHR and abdominoplasty SF-36 (physical)	5 [3] (± 3)	4 [4] (± 3)	0.57
Post IHR and abdominoplasty SF-36 (mental)	9 [9.5] (± 6)	12 [14] (± 5)	0.063
Pre RYGB DAS24	77.8 [80.5] (± 14)	73 [74] (± 14.5)	0.3
Pre IHR and abdominoplasty DAS24	45.1 [44.5] (± 7.22)	44 [44] (± 9)	0.63
Post IHR and abdominoplasty DAS24	24.1 [22.5] (± 5.8)	28 [26] (± 6)	0.026

The differences related to BMI decrease or changes in self-assessment by patients after surgery are statistically significant (*p* < 0.0001).

*Group 1* Prior to RYGB the average BMI in our patients was 53.68 (± 10.15). After RYGB the mean BMI decreased to 30 (± 3.27) (decrease of about 44.11%; *p* < 0.0001). Sublay IHR and abdominoplasty’s impact on the average BMI was definitely smaller, yet still statistically significant (decrease of 5.43%; *p* < 0.0001). Secondary BMI increase, 3–5 years after RYGB (Table 1), was seen in 75% (*n* = 15) of patients from the examined group. Five patients (25%) did not have a BMI change in the long-term follow-up when compared with the score post IHR with abdominoplasty. The increase of BMI was statistically significant (*p* = 0.002) with the average shift of 4.33. Therefore, the mean increase of 15.16% (28.37 vs. 32.67) was observed. In summary, mean BMI in the long-term follow-up was 32.67, median BMI 33.3, and standard deviation ± 5.12.

*Group 2* Prior to RYGB the average BMI in our patients was 46.84 (± 4.63). After RYGB the mean BMI decreased to 29.56 (± 4.64) (decrease of about 37%; *p* < 0.0001). Onlay IHR and abdominoplasty’s impact on the average BMI was definitely smaller, yet still statistically significant (decrease of 4.7%; *p* < 0.0001). Secondary BMI increase, 3–5 years after RYGB (Table 1), was confirmed in 55% of patients from the examined group; nine patients (45%) did not have a BMI change in the long-term follow-up when compared with the score post IHR with abdominoplasty. The increase of BMI was statistically significant (*p* = 0.001) with the average shift of 2.27. Therefore, the mean increase of 8.13% (27.9 versus 30, 16) was observed. In summary, mean BMI in the long-term follow-up was 30.16, median BMI 30.3 and standard deviation ± 7.64.

Patients with weight regain of both groups clearly stated problems with self-control, healthy eating habits and lack of regular physical activity (Table 1).

The direct results of IHR and abdominoplasty, and postoperative course (Table 2) were satisfactory, which resulted in rapid recovery of patients from both groups, but the comparison of the parameters related to the operation demonstrated longer duration of procedure, of suction drainage, and of analgesic agents use, also longer time to mobilization, and to full oral diet in patients operated with sublay IHR and abdominoplasty, than onlay method. Two persons after onlay IHR and abdominoplasty with wound complications underwent secondary wound suturing. The hospital stay was one month, complicated with pneumonia. The history of diabetes and hypertension was positive in these patients, and BMI scores before IHR and abdominoplasty were 35.4 kg/m^2^ and 40.4 kg/m^2^. Among three patients from the sublay IHR and abdominoplasty group with wound complications at the time of this surgery one had BMI 28.77 kg/m^2^, another 32.8 kg/m^2^ and the last one had positive history of diabetes and hypertension. All patients with confirmed bronchogenic pneumonia after sublay IHR and abdominoplasty were men.

Significant changes were also observed in self-assessment of patients from both groups.

**Fig. 1:**
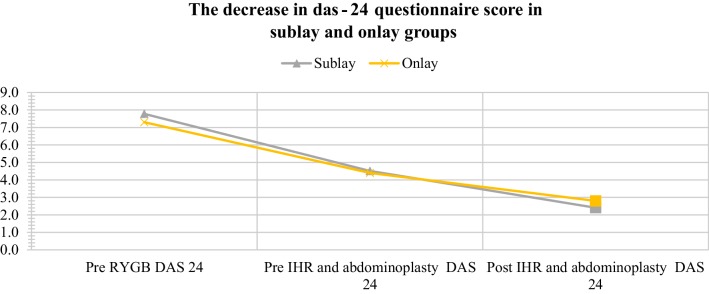
Decrease in the mean DAS24 questionnaire score in both groups. The difference between groups is statistically significant (*p* < 0.05) in the highlighted point

In the DAS24 scale the mean number of points decreased by 33 on average (by 42.3%) after RYGB operation and by next 21 points (by 46.67%) after sublay IHR and abdominoplasty (*p* < 0.0001 for both). On this scale, the mean number of points decreased by 29 on average (by 39.7%) after RYGB and by next 16 points (by 36.4%) after onlay IHR and abdominoplasty (*p* < 0.0001 for both). Aesthetically (DAS24), comparing to QL estimation before RYGB, we confirmed its improvement before abdominal contouring surgery 42% (sublay) and 39.7% (onlay), and after this operation 69% (sublay) and 61.6% (onlay) (Fig. 1).

**Fig. 2:**
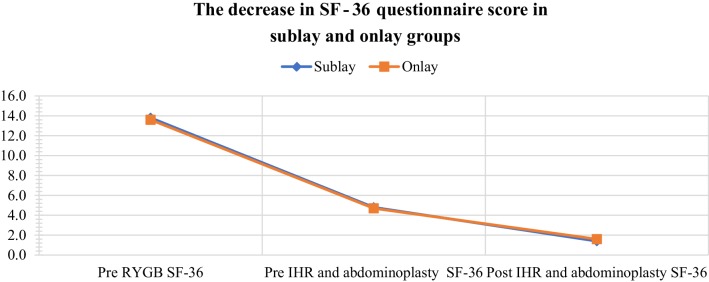
Decrease in the mean SF-36 questionnaire score in both groups. The differences between groups are not statistically significant (*p* > 0.005)

Regarding SF-36 scale, the mean decrease after RYGB was 90 points (around 65.22%; *p* < 0.0001) and after sublay IHR and abdominoplasty it decreased by further 34 points (by 70.83%; *p* < 0.0001). On this scale, the mean decrease after RYGB was 89 points (around 65.5%; *p* < 0.0001) and after onlay IHR and abdominoplasty it decreased by further 31 points (by 66%; *p* < 0.0001). Patient’s QL estimation in this scale before RYGB showed its general improvement before abdominal contouring surgery 65.2% (sublay), 65.4% (onlay), and after this operation 89.9% (sublay), 88.2% (onlay) (Fig. 2). Besides, the analysis of SF-36 questionnaire categories reveals a great score reduction in the physical category after sublay IHR and abdominoplasty (it decreased by 24 points, that is by 82.7%; *p* < 0.0001), and after onlay IHR and abdominoplasty it decreased by 24 points (that is by 85.7%; *p* < 0.0001). In contrast, the score decrease in the mental category was lesser, as the mean reduction was 10 points (decrease by 52.63%; *p* < 0.0001) after sublay IHR and abdominoplasty, and 7 points after onlay IHR and abdominoplasty (decrease by 36.84%; *p* < 0.0001). In this scale, patient’s QL estimation before RYGB improved before and after abdominal contouring surgery, respectively, 64.2% (sublay), 64.6% (onlay) and 93.9% (sublay) 94.9% (onlay)—in the physical dimension, 67.3% (sublay), 66.7% (onlay) and 84.5% (sublay) 78.9% (onlay)—in the mental dimension.

## Discussion

Body contouring operations can be complementary to surgical treatment in morbidly obese patients who underwent bariatric surgery, and after massive weight loss suffered from various body distortions at different body parts. Abdominoplasty is the most requested surgical procedure, as approximately 90% of patients after postoperative massive weight loss who come to plastic surgery undergo such intervention [10]. Combined abdominal wall operations allow to eliminate wound complications by moving fascial repair away from the skin incision site and removing redundant integument as a nidus for infection. In both our groups this allowed for the elimination of inflammations under the pendulum fold, observed before IHR and abdominoplasty in all individuals from both groups. This is consistent with observations of other authors [19]. In simultaneous IHR and abdominoplasty, the best option for mesh location and its tissue integration should be under consideration, as it may reduce hernia recurrence. This is more common in onlay and inlay repair, than sublay and underlay methods [18]. Mesh should have tissue coverage to minimize exposure to superficial as well as intra-peritoneal contents. However, wound complications, as well as mesh infections, increase the risk of hernia recurrence and are associated with higher rates of its repair failure or mesh rejection [11, 15]. The rate of wound complications (infection—7.3%, seroma/hematoma—6%) after paniculectomy is low, but increases according to other authors in patients after major weight loss and bariatric surgery [1, 20]. High frequency of incisional hernia after open bariatric procedures affects the frequency of simultaneous IHR and abdominoplasty in these groups of patients [1, 3, 16]. Some authors (Saxe et al.) state that these operations can be done together without significant additional morbidity, but opponents (Rubin et al.) are against simultaneous repair of very large hernias with other surgical procedures [12, 21]. Wound complication rate after sublay IHR abdominoplasty reported in Berry’s data was 8% (seroma—2%), and hernia recurrence—8% [11]. In our previous data during postoperative course with the use of this technique wound problems amounted up 16.6% (without seroma), but there was no hernia recurrence [3]. However, in a series of abdominal wall plication without mesh (Shermak’s data) wound problems were confirmed in 20% of cases, seroma in 12.5%, hernia recurred in 2.5% [13]. Borud reported minor and major wound complications in 50% of patients (seroma was noted in one case) and hernia recurrence in 8.5% after primary closure of hernia or together with abdominal wall plication in some cases with absorbable mesh onlay reinforcement [14]. Ortega, Saxe or Downey reported wound complication rates of approximately 40%, (without hernia recurrence), while Natarajan reported 15.4%, but seroma in 38.5%, hernia recurrence in 15.4% and mesh rejection in 30.8% after onlay IHR with abdominoplasty [12, 15, 16, 22]. Wound complications in the form of infection and abnormal healing we noted in the same rates (10%) in both groups, but without need of polypropylene mesh removal in any case. Two cases (10%) with mesh inserted with onlay method who demonstrated wound dehiscence required surgical reintervention, and monthly hospital stay; similar cases were reported in Ortega study [16]. We did not have any loss of navel, related by some authors to ventral hernia repair [12]. Seroma collection was observed only in cases with onlay mesh insertion (10%), which has been reported by other authors as a disadvantage of this technique [15]. Apart from that, we did not see hernia recurrences, nor mesh rejection in any case of our study with sublay as well as onlay mesh insertion during at minimum a 3-year follow-up.

Berry reported deep venous thrombosis or pulmonary embolus in 13% of his patients [11]. We did not experience any thromboembolic event, as preoperatively all patients in our study used thromboprophylaxis. Beneficial effects of prophylaxis was also confirmed by Saxe [12]. Bronchogenic pneumonia noted in our patients postoperatively undoubtedly was connected with limited function of respiratory muscles, and pulmonary atelectasis. Hematoma exfoliated the peritoneum in sublay IHR, may be a reason of impaired abdominal breathing track as well as prolonged pain (need for analgesic agents—12 (± 7) days). Men operated with this method are thus more prone to postoperative respiratory decompensation; in our study pneumonia after sublay IHR and abdominoplasty was seen only among men. Respiratory dysfunction was present in 10% of patients from the onlay group in both genders. They required prolonged hospital stay because of wound complications, and were additionally afflicted with diabetes and hypertension.

According to Vastine the rates of complictions are not related to BMI at the time of bariatric operation, but to abdominal wall surgery [23]. Ortega states that BMI at the time of surgery was not significantly different between complicated and uncomplicated patients in his data [16]. In Saxe report wound complication rate in patients with the history in diabetes and smoking was not higher comparing with patients without such history, and the author claims that weight loss has no impact on the severity of diabetes at the time of panniculectomy [12]. In our data two patients who underwent onlay IHR and abdominoplasty with wound complications had diabetes and hypertension, and their BMI before this surgery was 35.4 kg/m^2^ and 40.4 kg/m^2^. Among two cases after sublay IHR and abdominoplasty with wound complications at the time of this surgery one had BMI 28.77 kg/m^2^, another 32.8 kg/m^2^ and the last one had positive history of diabetes and hypertension.

The majority of reports related to simultaneous abdominal wall operations involve complications, but in turn are very selective in presenting postoperative course. Average operative time with the use of sublay technique in our data was 2.77 [3] (± 0.5) hours, while Berry reported 5.2 ± 0.2 h. Hospital stay of patients in our group was 10 [8.5] (± 3) days, similar to Berry’s report—9.0 ± 1.3 days [11]. Duration of drainage, according to Natarajan, varied between 3 (preperitoneal IHR) and 6 (onlay IHR) postoperative days, whereas in Ortega report 9–10 days (after onlay IHR) independently from very short (2–3 days) hospital stay [15, 16]. For comparison, duration of drainage in our patients after sublay IHR was 7 [7] (± 2) days, and for cases operated with onlay technique 5 [4] (± 1). Ortega, Shermak and Borud reported the same hospital stay of 3 days after primary IHR with abdominal wall plication, and similarly Saxe in groups of IHR without and with mesh (but mesh location was not mentioned) [12–14, 16]. He also quoted the length of surgery: 173 min and 204 min, respectively. Our data confirmed longer duration of procedure, of suction drainage, and of analgesic agents use, as well as longer time to mobilization and to full oral diet in patients operated with sublay IHR and abdominoplasty, as compared with onlay IHR and abdominoplasty. Rapid convalescence of patients both groups after this operation was related to length of abdominal contouring surgery, onset of oral feeding, patients mobilization, duration of drainage, and use of painkillers.

Some literature systematic reviews reported outcomes comprising different sections of improved QL as well as patient’s satisfaction and at the same time application of varied scales following body contouring surgery in patients after massive weight loss [24–27]. In our patients better QL was confirmed in respect of functional, psychological, and esthetic aspects after weight loss following RYGB, followed by further significant improvement after IHR and abdominoplasty in patient’s estimations. Our results correspond with the observations of others. Menderes also reported gradual improvement of general self-consciousness and self-consciousness of appearance in patients who followed bariatric surgery and then body contouring procedures [28]. Whereas some others (as Song) reporting poor QL (in HRQOL/SF-36) before bariatric surgery and its improvement after BMI loss, demonstrated very small improvement in patient’s opinions after body contouring (including abdominal procedures) [29]. Undoubtedly, simultaneity of IHR and abdominoplasty, as a single operation, and appropriate convalescence of patients from the examined groups in our study, contributed to significantly higher QL rating in comparison with the state following weight loss after RYGB.

Weight regain altogether was confirmed by 65% of patients (55% of patients from the onlay group and 75% from the sublay group) between the third and fifth year after RYGB, and resulted in their opinion from improper self-control, mainly eating habits, and periodically lack of physical activity, which corresponds with our earlier and other authors’ observations [3, 4]. This points to the need of constant and systematic postoperative follow-up by specialists dealing with weight reduction in bariatric groups, independently from the stage of their observation.

Presented here two methods of IHR and abdominoplasty are varied in respect of polypropylene mesh insertion. Onlay IHR, as less technically demanding, is connected with shorter operative time and shorter recovery. Sublay mesh placement poses smaller risk of wound and mesh complications or hernia recurrence. Both simultaneous techniques of IHR and abdominoplasty provide an opportunity to improve QL for patients after major weight loss, following open bariatric operations. Qualification for surgery and the choice of method should be individual in any case, depending on the general patient’s status and size of abdominal wall distortion, as well as patient’s expectations. Based on our experience, we recommend onlay IHR in larger of incisional hernias and abdominal wall distortions as less burdensome method for the patients especially with concomitant systemic (circulatory, respiratory) disorders, which can be also exasperated with age. Sublay method is safe in younger individuals, with less extensive incisional hernia and smaller abdominal integument requiring removal.

## Conclusions

One stage incisional hernia repair by onlay as well as sublay method with abdominoplasty are safe surgical methods improving the functioning of patients after major weight loss following RYGB. Sublay hernia repair and abdominoplasty was connected with longer time of the: operation, drainage, analgesic agents use, time to mobilization and to full oral diet than the onlay method. Significant improvement of the quality of life was noted after every subsequent step of surgical treatment in both groups. Reduction of the risk of BMI re-growth after bariatric surgery is related to the need for constant, specialized care for these patients at every stage of follow-up after bariatric surgery.
